# Tunneling Nanotube-Mediated Mitochondrial Transfer Rescues Nucleus Pulposus Cells from Mitochondrial Dysfunction and Apoptosis

**DOI:** 10.1155/2022/3613319

**Published:** 2022-03-04

**Authors:** Fan Yang, Yanbin Zhang, Sheng Liu, Jiheng Xiao, Yuxin He, Zengwu Shao, Yuhui Zhang, Xianyi Cai, Liming Xiong

**Affiliations:** ^1^Department of Orthopaedics, Union Hospital, Tongji Medical College, Huazhong University of Science and Technology, Wuhan 430022, China; ^2^Britton Chance Center and MoE Key Laboratory for Biomedical Photonics, Wuhan National Laboratory for Optoelectronics-Huazhong University of Science and Technology, Wuhan 430074, China

## Abstract

Stem cell-based therapy has been indicated to be beneficial for intervertebral disc regeneration. However, the underlying mechanisms have not been fully identified. The present study showed that bone marrow mesenchymal stem cells (BMSCs) donated mitochondria to adjacent nucleus pulposus cells (NPCs) in a coculture system. The mode of mitochondrial transfer between these cells was intercellular tunneling nanotube (TNT), which acted as a transportation expressway for mitochondria. NPCs acquired additional mitochondria from BMSCs in a concentration-dependent manner after rotenone-induced mitochondrial dysfunction in NPCs. Further research demonstrated that TNT-mediated mitochondrial transfer rescued NPCs from mitochondrial dysfunction and apoptosis, which was indicated by the recovery of the mitochondrial respiratory chain, the increase in mitochondrial membrane potential, and the decreases in reactive oxygen species (ROS) levels and apoptosis rates. Furthermore, Miro1, a critical protein that regulates mitochondrial movement, was knocked down in BMSCs and significantly reduced mitochondrial transfer from BMSCs to NPCs. These results suggested that Miro1 depletion inhibited the rescue of NPCs with mitochondrial dysfunction. Taken together, our data shed light on a novel mechanism by which BMSCs rescue impaired NPCs, providing a concrete foundation to study the critical role of intercellular interactions in disc regeneration.

## 1. Introduction

Stem cell-based therapy has great potential for the treatment of certain diseases [1]. Mesenchymal stem cells (MSCs) have unique immunosuppressive properties and self-renewal and transdifferentiation abilities. MSCs may be used as candidate cells for disease treatment. MSCs can be obtained from the bone marrow (BMSCs), adipose tissue, and other sources. A series of clinical trials confirmed the beneficial effects of MSCs [2–4]. MSC-based tissue engineering is the main research direction for the regeneration of intervertebral discs [5, 6]. Despite wide study, the mechanism underlying stem cell-based therapy is not understood. Intercellular interactions have attracted increasing attention as a novel mechanism underlying this therapy, and the transfer of organelles between cells has become the focus of many studies [7–9].

Mitochondria are highly dynamic double-membraned organelles that are present in most eukaryotic cells [10]. Mitochondria regulate intracellular homeostasis by controlling calcium signaling, energy production, and cell metabolism [11, 12]. Mitochondria are the power sources of cells and effectively produce ATP and a small number of destructive oxidants known as reactive oxygen species (ROS) [13–15]. Mitochondrial dysfunction is associated with some degenerative diseases, such as Alzheimer's disease, Parkinson's disease, and ischemic heart disease [16–18]. There is growing evidence indicating that MSCs donate mitochondria to a variety of cells, including endothelial cells, cardiomyocytes, and epithelial cells, to repair tissue damage [19–21]. The means of mitochondrial transfer involve tunneling nanotubes (TNTs), extracellular vesicles, and cellular fusion [22]. As a main mode of mitochondrial transfer, TNT is a significant and efficient cell-to-cell communication pathway that mediates intercellular interactions [23]. TNTs were first described in cultured pheochromocytoma cells [24], and subsequent studies showed that these structures connected many types of cells [25–27]. TNTs are membrane protuberances supported by actin polymer skeletons, but their phenotypes vary. For example, TNTs in macrophages contain only actin, and TNTs in other cells generally contain actin and microtubules [28]. There are two main mechanisms of TNT formation, and both mechanisms require cell movement and cytoskeletal remodeling. One mechanism involves filamentous extension from one cell to another cell, and the membrane fuses to form a TNT structure [29]. In the second mechanism, the membrane fuses between cells, and the migration of the cells in opposite directions lengthens the TNTs [30]. TNTs selectively transport a variety of cellular cargoes, including organelles, microRNAs, and vesicles [31]. TNTs can also transfer pathogens, such as bacteria and viruses, which leads to the corresponding pathological transmission [32, 33].

BMSCs can donate mitochondria to recipient cells through TNTs, thereby rescuing recipient cells [34, 35]. However, whether BMSCs donate mitochondria to nucleus pulposus cells (NPCs) with mitochondrial dysfunction via TNTs in intervertebral discs to rescue the impairment is not known. Moreover, mitochondrial Rho GTPase 1 (Miro1, also known as Rhot1) is a critical protein that regulates mitochondrial movement [36]. Miro1 helps mitochondria move along microtubules inside cells in the presence of a group of auxiliary proteins. When the expression of Miro1 in stem cells is increased, mitochondrial transfer is increased, and mitochondrial function in epithelial cells improves [36]. However, the key targets that regulate mitochondrial transfer between BMSCs and NPCs have not been elucidated.

Here, we investigated TNT-mediated mitochondrial transfer from BMSCs to NPCs and the specific benefits conferred to NPCs with mitochondrial dysfunction. In addition, we also studied the regulatory role of Miro1 in this process and its effect on the rescue of NPCs.

## 2. Materials and Methods

### 2.1. Isolation and Culture of Primary NPCs and BMSCs

All experimental procedures were approved by the Institutional Animal Care and Use Committee at Tongji Medical College, Huazhong University of Science and Technology. NPCs were isolated from Sprague-Dawley rats (male, 250-300 g) as described previously [37, 38]. The cells were cultured in Dulbecco's modified Eagle's medium/Ham's F-12 (DMEM/F-12, Gibco, USA) supplemented with 10% fetal bovine serum (FBS, Gibco, USA) and 1% penicillin-streptomycin (Sigma) at 37°C and 5% CO_2_. The medium was replaced every two days. The cells were expanded until the second passage. When the cells reached 80-90% confluency, second-generation NPCs were used for subsequent experiments. The same rats were used to isolate and obtain BMSCs simultaneously. The BMSCs were maintained in DMEM/F-12 (Gibco, USA) containing 10% FBS (Gibco, USA) supplemented with 1% penicillin-streptomycin (Sigma) at 37°C with 5% CO_2_. NPCs and BMSCs in the coculture system were derived from the same source.

### 2.2. Establishment of a Mitochondrial Dysfunction Model and a Coculture System *In Vitro*

To establish the mitochondrial dysfunction model, NPCs were treated with 2 *μ*M, 5 *μ*M and 10 *μ*M rotenone (rot, Sigma) for 2 h. The control group was treated with the same amount of DMSO. Then, we used 10 *μ*M rot to induce mitochondrial dysfunction in NPCs. To examine mitochondrial transfer between BMSCs and NPCs, cells were cocultured at a ratio of 1 : 1 in 6-well plates for 24 h. Transwell assays were also performed.

### 2.3. Cell Labeling

Mitochondria in BMSCs and NPCs were stained with 300 nM MitoTracker Red CMXRos (M7512, Invitrogen, USA) for 40 min at 37°C. NPCs were stained with 10 *μ*M carboxyfluorescein diacetate succinimidyl ester (CFSE, #C1031, Beyotime, China) for 30 min at 37°C. The cells were washed three times with phosphate-buffered saline (PBS). MitoTracker Red-stained cells and CFSE-stained NPCs were cocultured in medium for 24 h. Then, the cells were fixed with paraformaldehyde for 10 min. Phalloidin (AC18L012, Shanghai Life iLab Biotech, China) was used for F-actin staining lasting 45 min. Cellular fluorescence was captured using a confocal laser scanning microscope (FluoView FV3000, Olympus Corporation, Tokyo, Japan). ImageJ software (RRID: SCR_003070) was used for data analyses.

### 2.4. Treatment Groups

To assess the beneficial effect of mitochondrial transfer via TNTs on rot-induced NPCs, NPCs were exposed to 10 *μ*M rot for 2 h. Then, rot-induced NPCs were cocultured with BMSCs. The TNT inhibitor cytochalasin B (CB, 10 *μ*M, Sigma) was used in the coculture system the entire time. To evaluate the role of Miro1 in mitochondrial transfer, rot-induced NPCs were cocultured with BMSCs in which Miro1 had been knocked down.

### 2.5. Flow Cytometry and Cell Sorting

CellTrace Far Red (C34564, Thermo Fisher Scientific) was used to label the cytoplasm of rot-induced and untreated NPCs. Then, labeled NPCs were cocultured with BMSCs for 24 h. The mitochondrial membrane potential, the ROS level, and the apoptosis rate were analyzed by flow cytometry (Becton Dickinson, Franklin Lakes, NJ, USA), and CellTrace Far Red+ NPCs in the coculture system were identified as the detection objects. CellTrace Far Red+ NPCs was sorted by fluorescence-activated cell sorting (FACS) for subsequent experiments, such as the levels of apoptosis-related proteins, ATP, ADP, NADH, and NAD+.

### 2.6. JC-1 Staining

Mitochondrial membrane potential was detected by flow cytometry using a JC-1 (5,5′,6,6′-tetrachloro-1,1′,3,3′-tetraethyl-imidacarbocyanine iodide) staining kit (Beyotime, China) as described previously [39]. Briefly, the cells were harvested and incubated with 1 ml of JC-1 staining fluid for 20 min. After the cells were washed twice with PBS, the mitochondrial membrane potential was measured by flow cytometry and was expressed as the ratio of the red/green fluorescence intensity.

### 2.7. Measurement of ROS Levels

ROS levels were determined by the ROS assay kit (Beyotime, China). Cells were treated, harvested, and incubated with DCFH-DA for 20 min. The cells were washed three times with DMEM/F-12, and ROS levels were analyzed by flow cytometry.

### 2.8. Annexin V/Propidium Iodide (PI) Staining

Cell apoptosis was measured by Annexin V/PI staining (KeyGen Biotech, China) as described previously [15, 39]. The cells were treated, harvested, and resuspended in 500 *μ*l of binding buffer, and 5 *μ*l of Annexin V and 5 *μ*l of PI were added. Cell apoptosis was analyzed using flow cytometry.

### 2.9. Western Blot Analysis

Total protein was extracted from cells using RIPA lysis buffer (Beyotime, China). The protein concentrations were determined using a BCA protein assay kit (Beyotime, China). Equal amounts (30 *μ*g) of protein were resolved on a 10%-12% SDS-PAGE gel and then transferred onto PVDF membranes (Millipore, Burlington, MA, USA). The membranes were blocked with 5% nonfat milk and then incubated overnight at 4°C with primary antibodies against cleaved caspase-9 (1 : 5000, Abcam, USA, ab32539), Bax (1 : 1000, Affinity, AF0120), cleaved caspase-3 (1 : 500, Abcam, USA, ab32042), Bcl-2 (1 : 1000, Proteintech, 26593-1-AP), Miro1 (1 : 500, Abcam, USA, ab188029), and GAPDH (1 : 5000, Abcam, USA, ab8245). After being washed with Tris-buffered saline plus Tween-20 (TBST) three times, the membranes were incubated with secondary antibodies for 60 min at room temperature. The enhanced chemiluminescence method was used to visualize the proteins. ImageJ software was used to quantify the band densities.

### 2.10. ATP, ADP, NADH, and NAD+ Measurements

The ATP level was measured by an ATP assay kit (A095-1-1, Nanjing Jiancheng Bioengineering Institute, China). The ADP level was determined using an ADP assay kit (JYM0724Ra, Wuhan Jiyinmei Biotechnology Co., Ltd., Wuhan, China) according to the manufacturer's protocol. The cellular levels of NADH and NAD+ were measured using a NAD+/NADH assay kit with WST-8 (Beyotime, China). The absorbance was measured using a microplate reader.

### 2.11. Knockdown of Miro1 in BMSCs

BMSCs were seeded in 6-well plates and transfected with adenovirus for 12 h according to the manufacturer's instructions (GeneChem Co., Ltd., Shanghai, China). Then, the transfected BMSCs were cultured for 48 h. Miro1 knockdown BMSCs (BMSCmiro^Lo^) and control adenovirus-transfected cells (BMSCmiro^Cc^) were used in subsequent experiments.

### 2.12. Quantitative Real-Time PCR

Total RNA was extracted from cells by TRIzol reagent (Takara, Japan) according to the manufacturer's protocol. cDNA synthesis was performed using an iScript cDNA Synthesis kit (Bio-Rad). iTaq Universal SYBR Green Supermix (Bio-Rad) was used for quantitative real-time PCR. Data were collected using CFX Manager Software (Bio-Rad). The 2^-*ΔΔ*Ct^ method was used to analyze relative gene expression levels. The primer sequences were as follows: Aggrecan forward, 5′- GCAGCACAGACACTTCAGGA -3′; Aggrecan reverse, 5′- CCCACTTTCTACAGGCAAGC -3′; Sox9 forward, 5′- GGATGTCAAAGCAACAGGCG -3′; Sox9 reverse, 5′- ATGTGCGTTCTCTGGGACTG -3′; MMP13 forward, 5′- GTGACAGGAGCTAAGGCAGA -3′; MMP13 reverse, 5′- AGCATGAAAGGGTGGTCTCA -3′; Rhot1 forward, 5′- CCACTCTGTTTCCGTCACT -3′; Rhot1 reverse, 5′- CGCTACCTGTTTCTGCCT -3′; GAPDH forward, 5′- AAGTTCAACGGCACAGTCAA -3′; and GAPDH reverse, 5′- TCTCGCTCCTGGAAGATGG -3′.

### 2.13. Statistical Analysis

GraphPad Prism (GraphPad Software Inc., La Jolla, CA, USA) was used for statistical analysis. The data are expressed as the mean values ± standard deviation (SD). Student's *t*-test, one-way analysis of variance (ANOVA) with the Bonferroni post hoc test, and two-way ANOVA were used to determine the statistical significance of the differences between groups. A value of *P* < 0.05 was considered statistically significant.

## 3. Results

### 3.1. BMSCs Donate Exogenous Mitochondria to NPCs in a Coculture System

**Figure 1:**
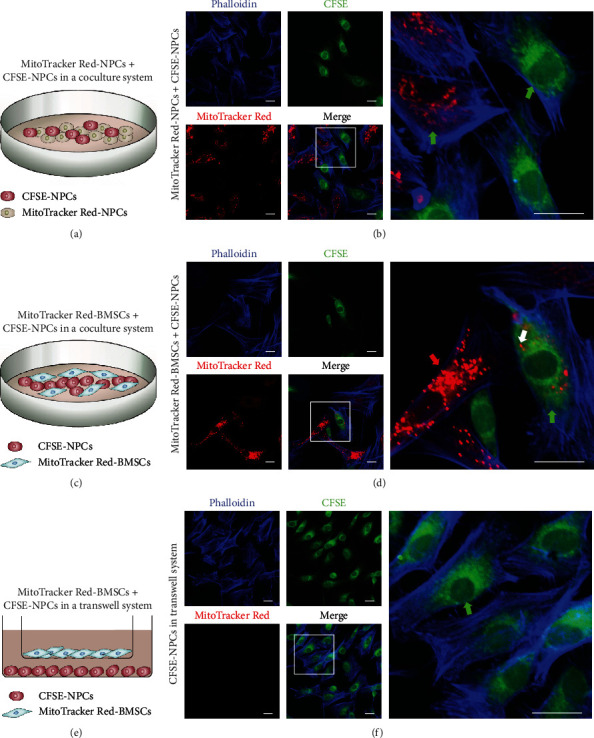
BMSCs donate exogenous mitochondria to NPCs in a coculture system. (a, b) Coculture of MitoTracker Red+ NPCs and CFSE+ NPCs. (c, d) Coculture of MitoTracker Red+ BMSCs and CFSE+ NPCs. (e, f) Transwell assay examining mitochondrial transfer between MitoTracker Red+ BMSCs and CFSE+ NPCs. Green arrowheads: NPCs; red arrowheads: BMSCs; white arrowheads: mitochondria. Scale bar, 20 *μ*m. BMSCs: bone marrow mesenchymal stem cells; NPCs: nucleus pulposus cells.

To investigate mitochondrial transfer from BMSCs or NPCs to adjacent NPCs, CFSE+ NPCs were cocultured with MitoTracker Red+ BMSCs or NPCs for 24 h. Confocal laser scanning microscopy was used to observe intercellular mitochondrial transfer. MitoTracker Red+ mitochondria from NPCs were detected in a few CFSE+ NPCs (Figures 1(a) and 1(b)). Notably, mitochondria were frequently observed in CFSE+ NPCs when MitoTracker Red+ BMSCs were used as donors in the coculture system (Figures 1(c) and 1(d)). In addition, we separated MitoTracker Red+ BMSCs and CFSE+ NPCs in a Transwell system, and almost no MitoTracker Red+ NPCs were detected (Figures 1(e) and 1(f)). These results demonstrate that BMSCs are more capable of providing exogenous mitochondria to NPCs than neighboring NPCs in a coculture system.

### 3.2. TNTs between BMSCs and NPCs in the Coculture System Effectively Mediate Intercellular Mitochondrial Transfer

**Figure 2:**
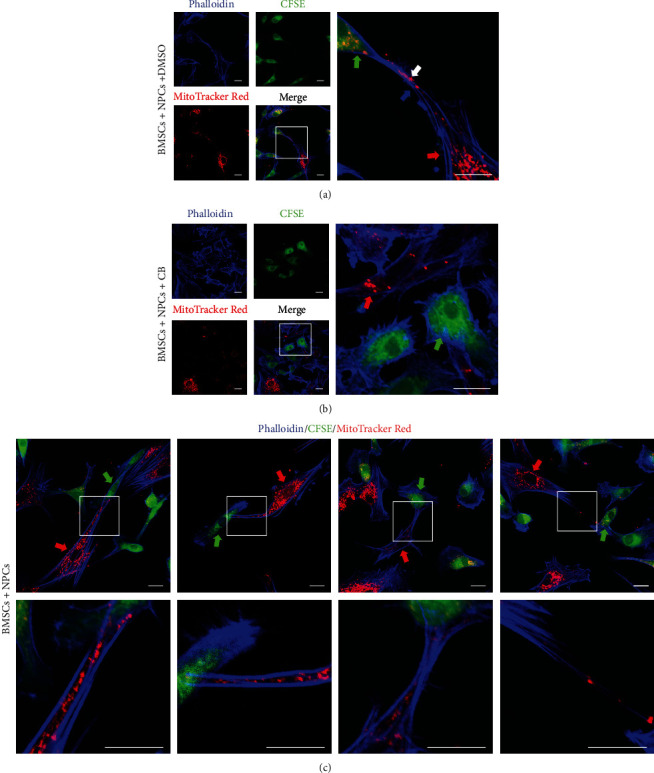
TNTs between BMSCs and NPCs in the coculture system effectively mediate intercellular mitochondrial transfer. (a) TNTs were observed between MitoTracker Red+ BMSCs and CFSE+ NPCs after the cells were cocultured for 24 h. (b) The formation of TNTs between MitoTracker Red+ BMSCs and CFSE+ NPCs after the cells were cocultured for 24 h in the presence of CB. (c) Different morphologies of TNTs that mediated mitochondrial transfer between BMSCs and NPCs. Green arrowheads: NPCs; red arrowheads: BMSCs; white arrowheads: mitochondria; blue arrowheads: TNTs. Scale bar, 20 *μ*m. BMSCs: bone marrow mesenchymal stem cells; CB: cytochalasin B; NPCs: nucleus pulposus cells; TNTs: tunneling nanotubes.

We further investigated the means of mitochondrial transfer between donor and recipient cells. Confocal laser scanning microscopy showed F-actin-positive tubular microstructures between BMSCs and NPCs, and TNTs mediated the intercellular mitochondrial transfer (Figure 2(a)). To examine whether NPCs acquired mitochondria from BMSCs with TNT deficiency, cells were treated with CB to induce F-actin aggregation and inhibit the formation of TNTs. Almost no TNT formation was detected between BMSCs and NPCs, and few MitoTracker Red+ mitochondria were observed in NPCs (Figure 2(b)). Notably, the morphology of TNTs between cells was diverse, which increased the diversity of mitochondrial transfer (Figure 2(c)). Briefly, TNTs between BMSCs and NPCs in the coculture system allow for effective intercellular mitochondrial transfer, which suggests that TNTs are essential for mitochondrial transfer.

### 3.3. BMSCs Can Donate Additional Mitochondria to NPCs with Mitochondrial Dysfunction

**Figure 3:**
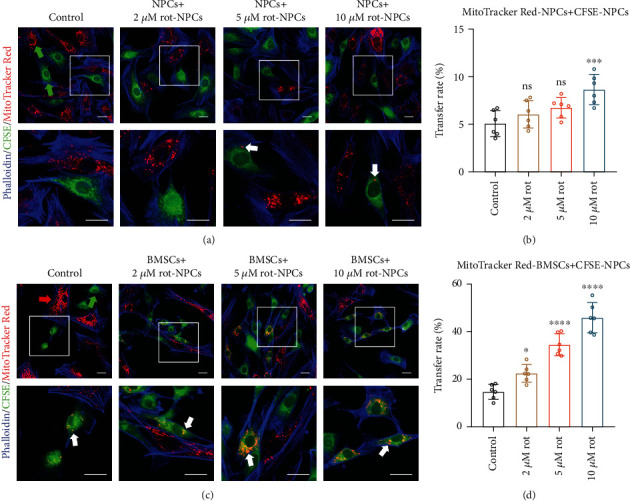
BMSCs can donate additional mitochondria to NPCs with mitochondrial dysfunction. (a, b) Representative confocal images and histogram analysis showing that CFSE+ NPCs pretreated with 0, 2 *μ*M, 5 *μ*M, and 10 *μ*M rot received mitochondria from neighboring MitoTracker Red+ NPCs and the mitochondrial transfer rate. (c, d) Representative confocal images and histogram showing that CFSE+ NPCs pretreated with 0, 2 *μ*M, 5 *μ*M, and 10 *μ*M rot received mitochondria from neighboring MitoTracker Red+ BMSCs and the mitochondrial transfer rate. Green arrowheads: NPCs; red arrowheads: BMSCs; white arrowheads: mitochondria. Scale bar, 20 *μ*m. Means ± SD, *n* = 6. ^∗^*P* < 0.05, ^∗∗∗^*P* < 0.001, and ^∗∗∗∗^*P* < 0.0001, ns: not significant vs. the control group. BMSCs: bone marrow mesenchymal stem cells; NPCs: nucleus pulposus cells; rot: rotenone.

To further examine whether recipient cells can take up additional exogenous mitochondria when their mitochondria were impaired, we used rot, a respiratory chain complex I inhibitor, to induce mitochondrial dysfunction. However, as the rot concentration increased, the mitochondrial transfer between MitoTracker Red+ NPCs and CFSE+ NPCs did not increase significantly (Figures 3(a) and 3(b)). Instead, rot enhanced mitochondrial uptake by NPCs from BMSCs (Figures 3(c) and 3(d)). In contrast, there were few MitoTracker Red+ NPCs in the Transwell system with increasing rot concentration (Figure S1). These results demonstrate that BMSCs donate additional mitochondria to NPCs with mitochondrial dysfunction in a coculture system.

### 3.4. Mitochondrial Transfer Improves Mitochondrial Dysfunction in NPCs

**Figure 4:**
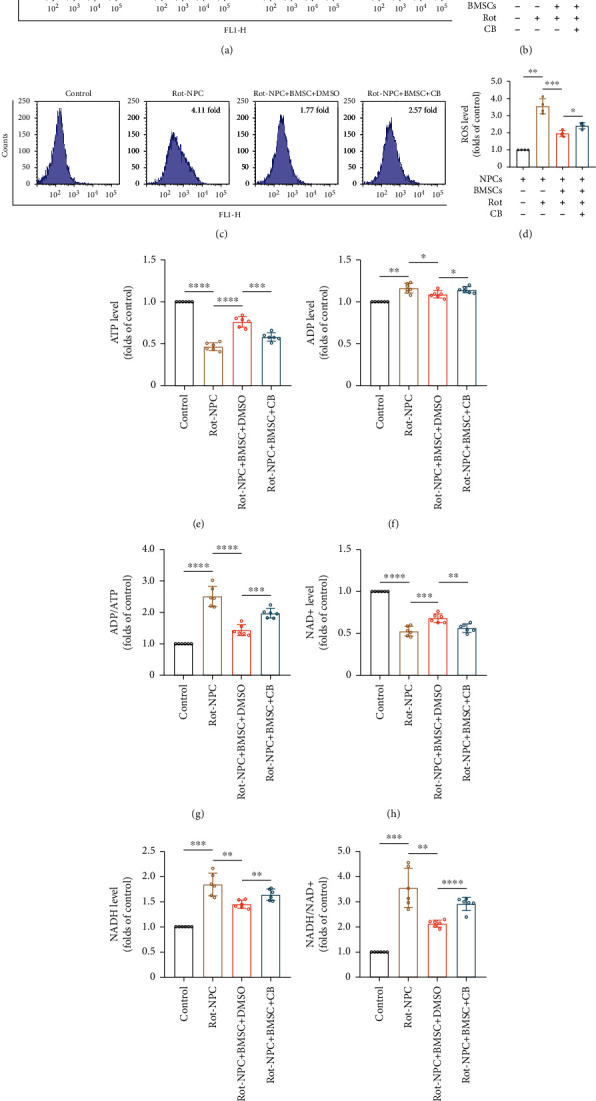
Mitochondrial transfer improves mitochondrial dysfunction in NPCs. (a) Flow cytometric analysis of mitochondrial membrane potential in NPCs. (b) Histogram showing the mitochondrial membrane potential. Means ± SD, *n* = 4. (c) Flow cytometric analysis of ROS levels in NPCs. (d) Relative levels of intracellular ROS. Means ± SD, *n* = 4. (e–g) Histogram showing the ATP, ADP, and ADP/ATP levels in NPCs. (h–j) Histogram showing the levels of NAD+, NADH, and NADH/NAD+ in NPCs. Means ± SD, *n* = 6. ^∗^*P* < 0.05, ^∗∗^*P* < 0.01, ^∗∗∗^*P* < 0.001, and ^∗∗∗∗^*P* < 0.0001. BMSCs: bone marrow mesenchymal stem cells; CB: cytochalasin B; NPCs: nucleus pulposus cells; rot: rotenone.

To assess the functional alterations of mitochondria in NPCs after receiving mitochondria from BMSCs, we examined mitochondrial membrane potential and ROS levels in NPCs. NPCs were prestained with CellTrace Far Red (Figure S2A). Mitochondrial damage decreased mitochondrial membrane potential, which was characterized by a decrease in the ratio of red/green fluorescence intensity. After the cells were cocultured with BMSCs, we observed an increase in the red/green ratio of NPCs, indicating that mitochondrial damage was partially relieved. The red/green ratio decreased after the TNT inhibitor CB was added to the medium (Figures 4(a) and 4(b)). We used DCFH-DA fluorescent probes to detect ROS levels in NPCs and demonstrated that ROS levels significantly increased in a rot concentration-dependent manner (Figure S3A-B). To examine whether mitochondrial transfer via TNTs prevented mitochondrial dysfunction in NPCs, NPCs were cocultured with BMSCs in the presence of CB. Rot-induced production of ROS in NPCs was significantly reduced after coculture, and CB inhibited these effects (Figures 4(c) and 4(d)). These results suggest that mitochondrial transfer via TNTs partially increases mitochondrial membrane potential and decreases the production of ROS.

To identify whether mitochondrial transfer prevented the inhibitory effect of rot on the respiratory chain, we analyzed intracellular ATP, ADP, NAD+, and NADH levels in FACS harvested CellTrace Far Red+ NPCs (Figure S2B). ATP and NAD+ levels decreased after NPCs were treated with 2 *μ*M, 5 *μ*M, and 10 *μ*M rot, while ADP and NADH levels increased compared to the control in a concentration-dependent manner (Figure S3C-H). As shown in Figures 4(e)–4(j), the levels of ATP and NAD+ in NPCs increased after the cells were cocultured with BMSCs as compared with the rot-treated group. In contrast, ADP, ADP/ATP, NADH, and NADH/NAD+ levels decreased. CB treatment partially suppressed these effects. Taken together, these data indicate that mitochondrial transfer via TNTs in the coculture system rescue the inhibitory effect of rot on the respiratory chain.

### 3.5. Effects of Mitochondrial Transfer on Apoptosis and Matrix Metabolism in NPCs

**Figure 5:**
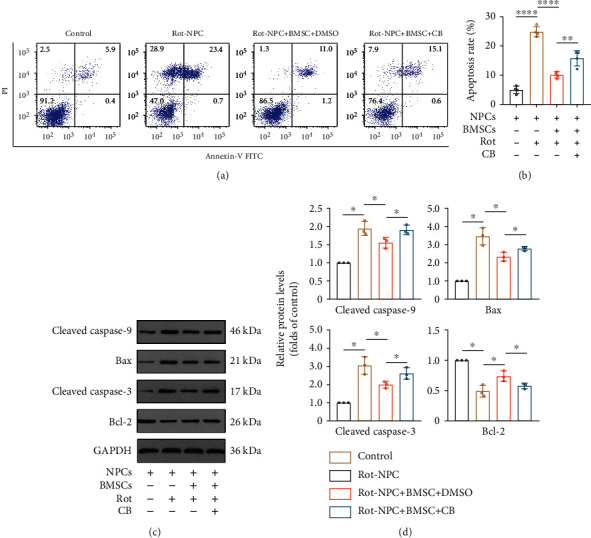
Mitochondrial transfer inhibits apoptosis in NPCs with mitochondrial dysfunction. (a) Flow cytometric analysis of the apoptosis rates of NPCs. (b) Histogram showing the apoptosis rate. Means ± SD, *n* = 4. (c) Representative Western blots showing the expression of cleaved caspase-9, Bax, cleaved caspase-3, and Bcl-2 in NPCs. (d) Relative protein levels of cleaved caspase-9, Bax, cleaved caspase-3, and Bcl-2. Means ± SD, *n* = 3. ^∗^*P* < 0.05, ^∗∗^*P* < 0.01, and ^∗∗∗∗^*P* < 0.0001. BMSCs: bone marrow mesenchymal stem cells; CB: cytochalasin B; NPCs: nucleus pulposus cells; rot: rotenone.

Mitochondrial dysfunction induced by rot may lead to apoptosis in NPCs. Annexin V/PI staining demonstrated that rot markedly increased the apoptosis rates of NPCs (Figure S4A-B). To examine whether rot-induced apoptosis could be prevented by mitochondrial transfer via TNTs, after coculturing with BMSCs, we observed a decrease in apoptosis rate of NPCs. Moreover, after adding the TNT inhibitor CB to the medium, the apoptosis rate increased (Figures 5(a) and 5(b)). Additionally, FACS was used to harvest CellTrace Far Red+ NPCs from the coculture system, and Western blotting was performed to examine the expression of apoptosis-related proteins, such as cleaved caspase-9, Bax, cleaved caspase-3, and Bcl-2. The expression of the antiapoptotic protein Bcl-2 decreased with increasing rot concentration, and the expression of the proapoptotic protein Bax, cleaved caspase-9, and cleaved caspase-3 increased (Figure S4C-D). The expression of cleaved caspase-9, Bax, and cleaved caspase-3 decreased, and the expression of Bcl-2 increased in NPCs after coculture with BMSCs. CB treatment partially inhibited these beneficial effects (Figures 5(c) and 5(d)). In addition, the effects of mitochondrial transfer on NPC matrix anabolism and catabolism were further studied. After coculturing with BMSCs, we observed an increase in Aggrecan and Sox9 expressions of rot pretreated NPCs and a decrease in matrix metalloproteinase 13 (MMP13) expression (Figure S5A-C). These results indicate that the intercellular mitochondrial transfer via TNTs showed some beneficial effects on apoptosis and matrix metabolism in NPCs.

### 3.6. Miro1 Knockdown Reduces Intercellular Mitochondrial Transfer

**Figure 6:**
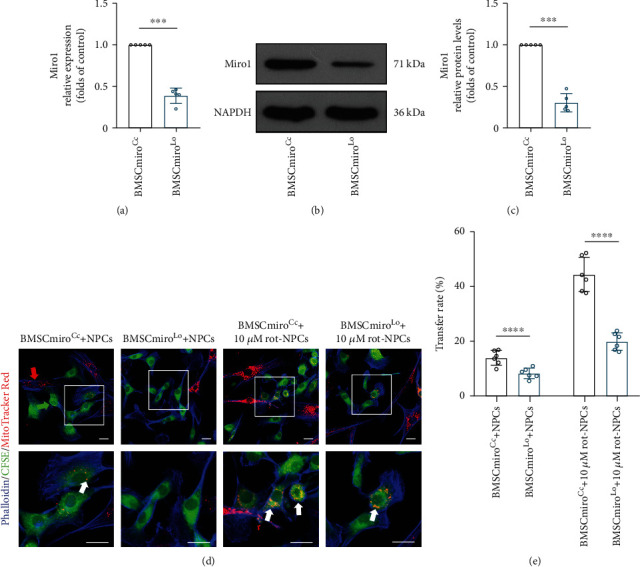
Miro1 knockdown reduces intercellular mitochondrial transfer. (a) Rhot1 knockdown efficiency was measured by quantitative real-time PCR. (b, c) Western blot confirming the efficient knockdown of Miro1. Means ± SD, *n* = 5. (d, e) Representative confocal images and histogram showing that CFSE+ NPCs received mitochondria from neighboring MitoTracker Red+ BMSCmiro^Cc^ and BMSCmiro^Lo^ and the mitochondrial transfer rate. Green arrowheads: NPCs; red arrowheads: BMSCs; white arrowheads: mitochondria. Scale bar, 20 *μ*m. Means ± SD, *n* = 6. ^∗∗∗^*P* < 0.001, ^∗∗∗∗^*P* < 0.0001. BMSCs: bone marrow mesenchymal stem cells; BMSCmiro^Cc^: control adenovirus-transfected BMSCs; BMSCmiro^Lo^: BMSCs transfected with adenovirus carrying Rhot1 shRNA; NPCs: nucleus pulposus cells; rot: rotenone.

To verify the functional role of Miro1 in mitochondrial transfer, we used adenovirus carrying Rhot1 shRNA to knockdown Miro1 expression in BMSCs (BMSCmiro^Lo^). We used adenovirus carrying nonspecific shRNA as a control (BMSCmiro^Cc^). Efficient Miro1 knockdown was validated using quantitative real-time PCR and Western blotting (Figures 6(a)–6(c)). We found that BMSCmiro^Lo^ donated fewer mitochondria to NPCs in the coculture system than BMSCmiro^Cc^. Augmentation of mitochondrial dysfunction in NPCs induced a smaller increase in the transfer rate compared to the control group (Figures 6(d) and 6(e)), which suggests that Miro1 depletion confers a disadvantage to mitochondrial transfer.

### 3.7. Miro1 Knockdown Reduces the Beneficial Effect of Mitochondrial Transfer on Impaired NPCs

**Figure 7:**
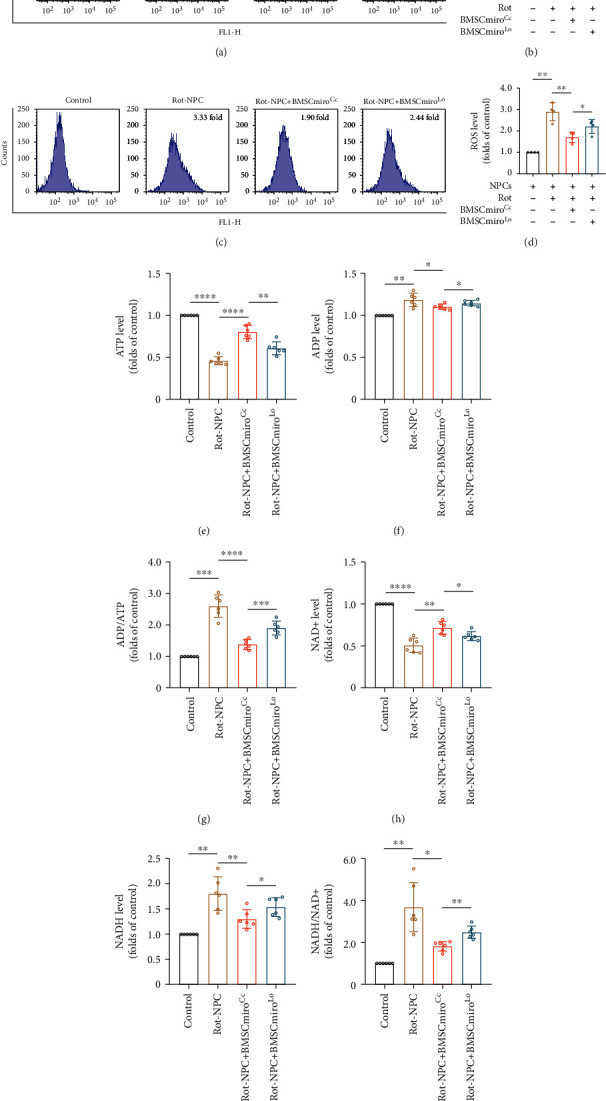
Miro1 knockdown decreases the efficiency of mitochondrial functional rescue in NPCs. (a) Flow cytometric analysis of mitochondrial membrane potential in NPCs cocultured with BMSCmiro^Lo^. (b) Histogram showing the mitochondrial membrane potential. Means ± SD, *n* = 4. (c) Flow cytometric analysis of ROS levels in NPCs cocultured with BMSCmiro^Lo^. (d) Relative levels of intracellular ROS. Means ± SD, *n* = 4. (e–g) Histogram showing the levels of ATP, ADP, and ADP/ATP in NPCs cocultured with BMSCmiro^Lo^. (h–j) Histogram showing the levels of NAD+, NADH, and NADH/NAD+ in NPCs cocultured with BMSCmiro^Lo^. Means ± SD, *n* = 6. ^∗^*P* < 0.05, ^∗∗^*P* < 0.01, ^∗∗∗^*P* < 0.001, and ^∗∗∗∗^*P* < 0.0001. BMSCs: bone marrow mesenchymal stem cells; BMSCmiro^Cc^: control adenovirus-transfected BMSCs; BMSCmiro^Lo^: BMSCs transfected with adenovirus carrying Rhot1 shRNA; NPCs: nucleus pulposus cells; rot: rotenone.

Because Miro1 may be essential for mitochondrial transfer, flow cytometry was used to quantitatively measure the mitochondrial function changes of impaired NPCs after cocultured with BMSCmiro^Lo^ and BMSCmiro^Cc^, such as the mitochondrial membrane potential and ROS levels. Compared to NPCs cocultured with BMSCmiro^Cc^, the red/green ratio of NPCs cocultured with BMSCmiro^Lo^ decreased, and the ROS levels increased (Figures 7(a)–7(d)). To further determine the regulatory role of Miro1 in the mitochondrial transfer between BMSCs and NPCs, we analyzed intracellular ATP, ADP, NAD+, and NADH levels in NPCs cocultured with BMSCmiro^Lo^. The results showed that the levels of ATP and NAD+ decreased, and ADP, ADP/ATP, NADH, and NADH/NAD+ levels increased compared to NPCs cocultured with BMSCmiro^Cc^ (Figures 7(e)–7(j)).

**Figure 8:**
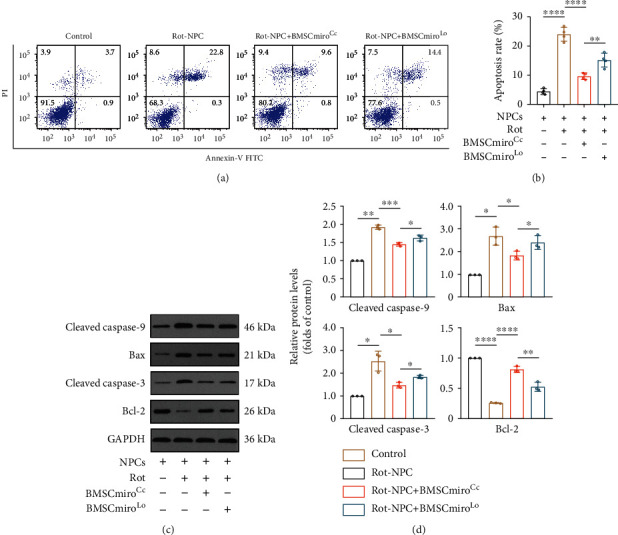
Miro1 knockdown decreases the efficiency of apoptosis rescue in NPCs. (a) Flow cytometric analysis of the apoptosis rates of NPCs cocultured with BMSCmiro^Lo^. (b) Histogram showing the apoptosis rate. Means ± SD, *n* = 4. (c) Representative Western blots showing the expression of cleaved caspase-9, Bax, cleaved caspase-3, and Bcl-2 in NPCs cocultured with BMSCmiro^Lo^. (d) Relative protein levels of cleaved caspase-9, Bax, cleaved caspase-3, and Bcl-2. Means ± SD, *n* = 3. ^∗^*P* < 0.05, ^∗∗^*P* < 0.01, ^∗∗∗^*P* < 0.001, and ^∗∗∗∗^*P* < 0.0001. BMSCs: bone marrow mesenchymal stem cells; BMSCmiro^Cc^: control adenovirus-transfected BMSCs; BMSCmiro^Lo^: BMSCs transfected with adenovirus carrying Rhot1 shRNA; NPCs: nucleus pulposus cells; rot: rotenone.

To further investigate whether Miro1 knockdown affected mitochondrial transfer-mediated resistance to NPC apoptosis, we used flow cytometry to measure the apoptosis rate and Western blotting to measure the expression of apoptosis-related proteins. Compared to NPCs cocultured with BMSCmiro^Cc^, NPCs cocultured with BMSCmiro^Lo^ showed a higher rate of apoptosis and expressed fewer antiapoptotic proteins and more proapoptotic proteins (Figures 8(a)–8(d)). Overall, Miro1 knockdown significantly reduced mitochondrial donation from BMSCs to NPCs, which reduced the beneficial effect on NPCs with mitochondrial dysfunction.

## 4. Discussion

Stem cell-based therapy in the treatment of intervertebral disc degeneration is promising, and the therapeutic efficacy may depend on paracrine and anti-inflammatory effects according to previous studies [40, 41]. Recent emerging evidence showed that intercellular interactions were essential in tissue repair. Research on MSC-derived exosomes has made great progress in disc regeneration [42, 43]. As another form of cell-to-cell interaction, mitochondrial transfer was rarely reported in intervertebral discs. One study suggested that mitochondrial transfer reduces the apoptosis rate of NPCs when they were exposed to inflammatory factor [44]. Compelling evidence in other fields showed that MSCs rescued ocular cells and lung epithelial cells via mitochondrial transfer [35, 36]. To further examine the interaction between BMSCs and NPCs in stem cell therapy, it is imperative to investigate mitochondrial transfer between these cells and the beneficial effect on NPCs. The regulatory mechanisms should also be understood. The present study showed that BMSCs donated mitochondria to NPCs, which was mediated by TNTs. Notably, mitochondrial dysfunction improved after NPCs with mitochondrial dysfunction received mitochondria from BMSCs, and the apoptosis rate decreased. We found that adenovirus-mediated knockdown of Miro1, which is a regulator of mitochondrial motility, abrogated mitochondrial transfer and significantly reduced the rescue effect.

Mitochondrial dysfunction and matrix metabolism imbalance are essential in the homeostasis, development, and pathogenesis of intervertebral discs. Mitochondrial dysfunction increases the production of ROS and leads to oxidative damage [45]. Mitochondrial dysfunction also causes apoptosis in NPCs and induces disc degeneration [46]. Matrix metabolism imbalance is bound up with intervertebral disc degeneration [47, 48]. NPCs in degenerative discs are prone to mitochondrial dysfunction and apoptosis, and mitochondrial transfer may be a novel means to solve this problem. The results of the present study indicated that BMSCs donated mitochondria to NPCs. NPCs received additional mitochondria from BMSCs with the exacerbation of mitochondrial dysfunction. This finding suggested that BMSCs responded to damage to NPCs in the coculture system and repaired the impaired NPCs. These results support mitochondrial transfer between BMSCs and NPCs as a potential method to repair mitochondrial dysfunction and ameliorate disc degeneration.

TNTs are intercellular junctions that transport a variety of cargoes ranging from ions to entire organelles. TNT-mediated mitochondrial transfer is beneficial for the homeostasis of recipient cells [36, 49]. The present study found that TNTs formed between BMSCs and NPCs *in vitro* and mediated intercellular mitochondrial transfer. TNTs between cells were present in the coculture system, and TNT morphology was diverse. An inhibitor of TNT formation, CB, significantly inhibited mitochondrial transfer between cells. This finding is consistent with previous reports [20, 35]. These data further demonstrate the importance of TNTs in the transfer of mitochondria between cells.

After establishing that BMSCs donate mitochondria to NPCs with mitochondrial dysfunction, we explored the beneficial effect of this process on impaired NPCs. Previous studies indicated that the mitochondrial function of endothelial cells and cardiomyocytes under stress improved after mitochondria were transferred from MSCs, and the cells were partially rescued [19, 20]. Therefore, it was critical to confirm the beneficial effect of mitochondrial transfer. Our results demonstrated that the mitochondrial membrane potential of NPCs with mitochondrial dysfunction increased after the cells were cocultured with BMSCs, the level of ROS and apoptosis rate decreased, matrix anabolism increased, and catabolism decreased. The rot-injured mitochondrial respiratory chain in recipient NPCs was partially repaired, which increased ATP levels. Notably, the beneficial effect on NPCs was significantly reduced after inhibiting the formation of TNTs in the coculture system. These results suggest that BMSCs rescue NPCs with mitochondrial dysfunction via mitochondrial transfer, and the formation of TNTs is closely related to this process.

The mechanism regulating mitochondrial transfer from BMSCs to NPCs was further examined. The mitochondrial adapter protein Miro1 regulates the movement of mitochondria. Miro1 overexpression promoted mitochondrial transfer between cells and improved the function of alveolar epithelial cells after injury [36]. The current study showed that BMSCmiro^Lo^ rarely donated mitochondria to NPCs. The beneficial effects on NPCs were significantly reduced. In summary, these results indicated that the downregulation of Miro1 significantly affected the beneficial effects of BMSCs on NPCs with mitochondrial dysfunction. Therefore, Miro1 is a potential target for regulating BMSC donation of mitochondria to NPCs and an attractive tool for dissecting intercellular interactions in intervertebral discs.

Our study has several limitations. First, we used rat BMSCs and NPCs for these experiments due to limited access to BMSCs and NPCs in healthy humans. Second, our study was performed *in vitro*, which does not directly reflect clinical settings. Therefore, further study using disease models and repair mechanisms *in vivo* is warranted. High-resolution real-time microscopic techniques, such as structured illumination microscopy, may be used to further observe the formation of TNTs and mitochondrial transfer between cells. Although we examined the regulatory role of the key protein Miro1 in mitochondrial transfer, the effects of other proteins, such as Miro2, TRAK1, TRAK2, Myo19, and TNFAIP2, on intercellular interactions in intervertebral discs require further study. rot, as an inhibitor of mitochondrial complex 1, cannot simulate the pathological environment of intervertebral disc degeneration. Whether mitochondrial transfer shows beneficial effects on NPCs under inflammatory factors or oxidative stress deserves further study.

**Figure 9:**
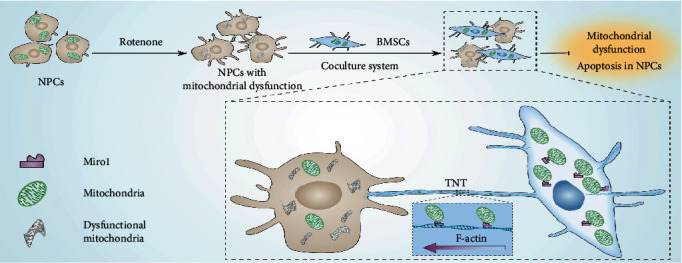
Schematic illustration showing that TNT-mediated mitochondrial transfer from BMSCs rescues NPCs with mitochondrial dysfunction.

In summary, the current study demonstrated that mitochondrial transfer from BMSCs via TNTs rescued NPCs with mitochondrial dysfunction and the key role of Miro1 in this process (Figure 9). These findings provide a concrete foundation to study the critical role of intercellular interactions in disc regeneration. Strategies targeting intercellular crosstalk are promising in the development of novel treatments for intervertebral disc degeneration.

## Data Availability

The data used to support the findings of this study are available from the corresponding author upon request.

## Supplementary Materials

Supplementary Materials Supplemental Figure 1: mitochondrial transfer from BMSCs to NPCs with mitochondrial dysfunction in a Transwell assay. Supplemental Figure 2: NPCs were identified by flow cytometry, and NPCs were harvested by FACS. Supplemental Figure 3: rot induces mitochondrial dysfunction in NPCs. Supplemental Figure 4: rot induces apoptosis in NPCs. Supplemental Figure 5: the effects of mitochondrial transfer on matrix anabolism and catabolism in NPCs.
