# Supplementary material for: Tunneling Nanotube-Mediated Mitochondrial Transfer Rescues Nucleus Pulposus Cells from Mitochondrial Dysfunction and Apoptosis
Source: Oxid Med Cell Longev. 2022 Mar 4;2022:3613319. doi: 10.1155/2022/3613319 (PMC8916857; doi:10.1155/2022/3613319)
Supplement: Supplementary Materials — Supplemental Figure 1: mitochondrial transfer from BMSCs to NPCs with mitochondrial dysfunction in a Transwell assay. Supplemental Figure 2: NPCs were identified by flow cytometry, and NPCs were harvested by FACS. Supplemental Figure 3: rot induces mitochondrial dysfunction in NPCs. Supplemental Figure 4: rot induces apoptosis in NPCs. Supplemental Figure 5: the effects of mitochondrial transfer on matrix anabolism and catabolism in NPCs. [file 3613319.f1.doc]

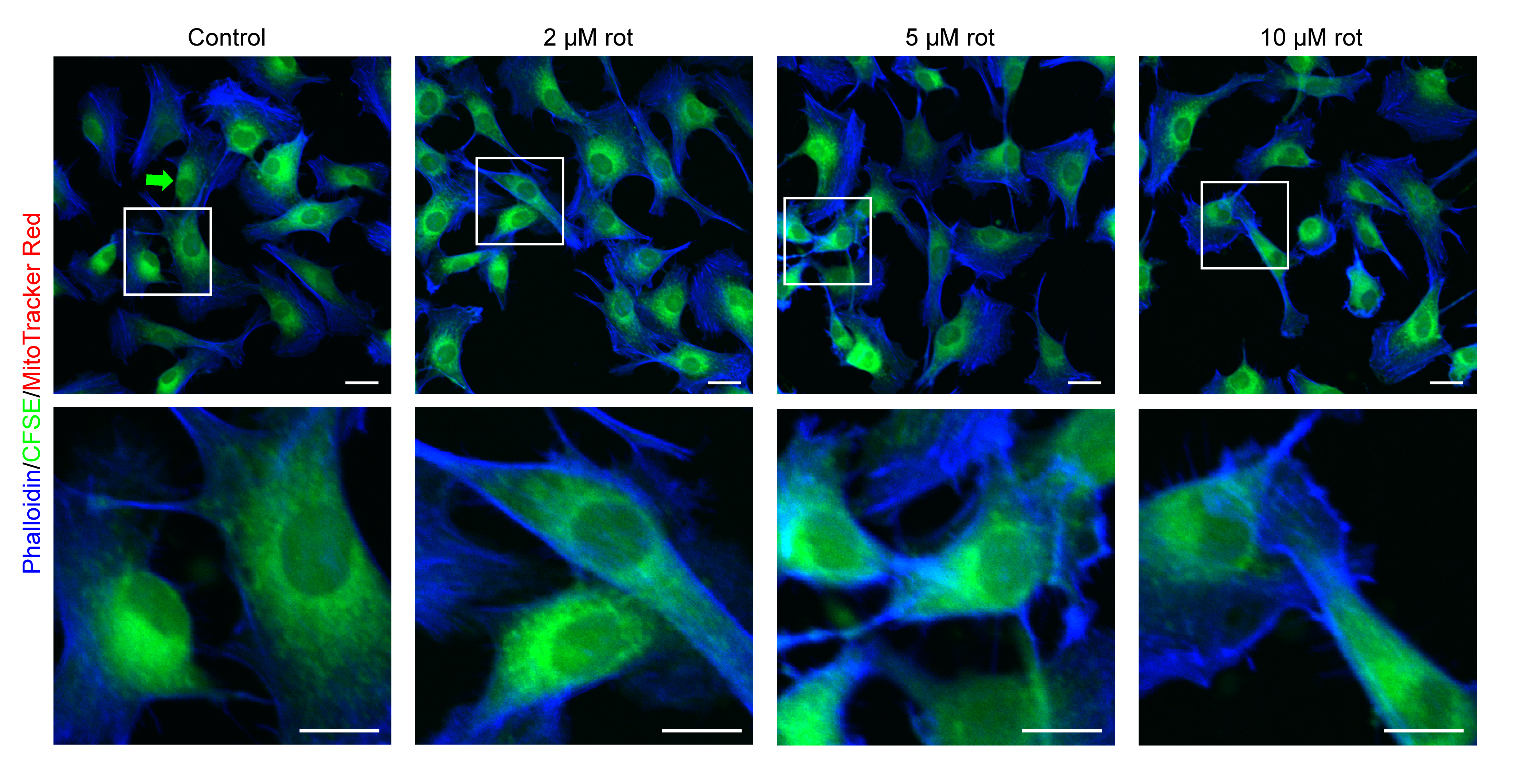


Supplemental Figure 1. Mitochondrial transfer from BMSCs to NPCs with mitochondrial dysfunction in a Transwell assay. Transwell assay examining mitochondrial transfer between MitoTracker Red+ BMSCs and CFSE+ NPCs pretreated with 0 µM, 2 µM, 5 µM, and 10 µM rot. Green arrowheads: NPCs. Scale bar, 20 μm. BMSCs, bone marrow mesenchymal stem cells; NPCs, nucleus pulposus cells; rot, rotenone.


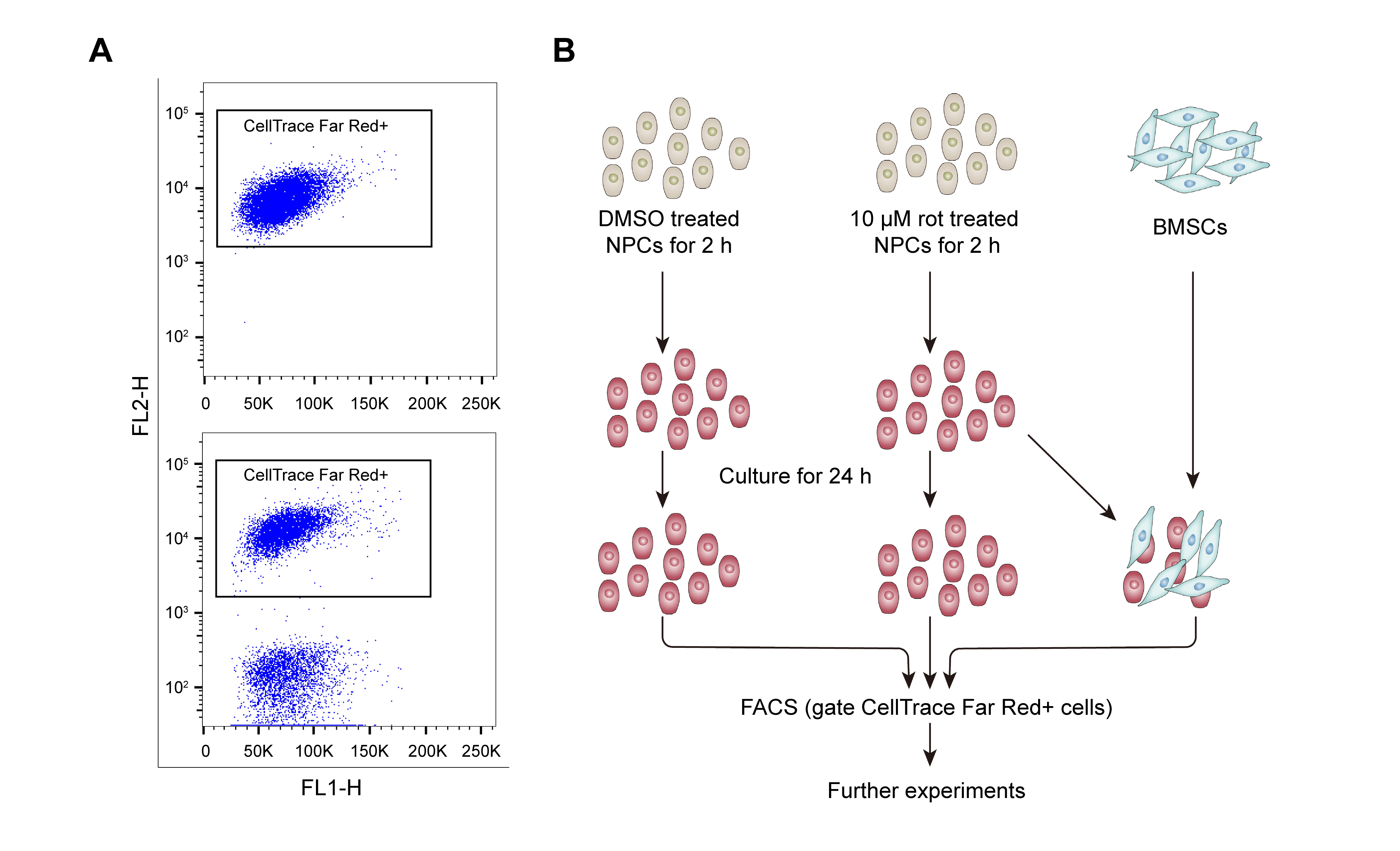


Supplemental Figure 2. NPCs were identified by flow cytometry, and NPCs were harvested by FACS. (A) Representative flow cytometric analysis of CellTrace Far Red+ NPCs. (B) CellTrace Far Red+ NPCs from the different groups were sorted by FACS for further experiments. BMSCs, bone marrow mesenchymal stem cells; FACS, fluorescence-activated cell sorting; NPCs, nucleus pulposus cells; rot, rotenone.


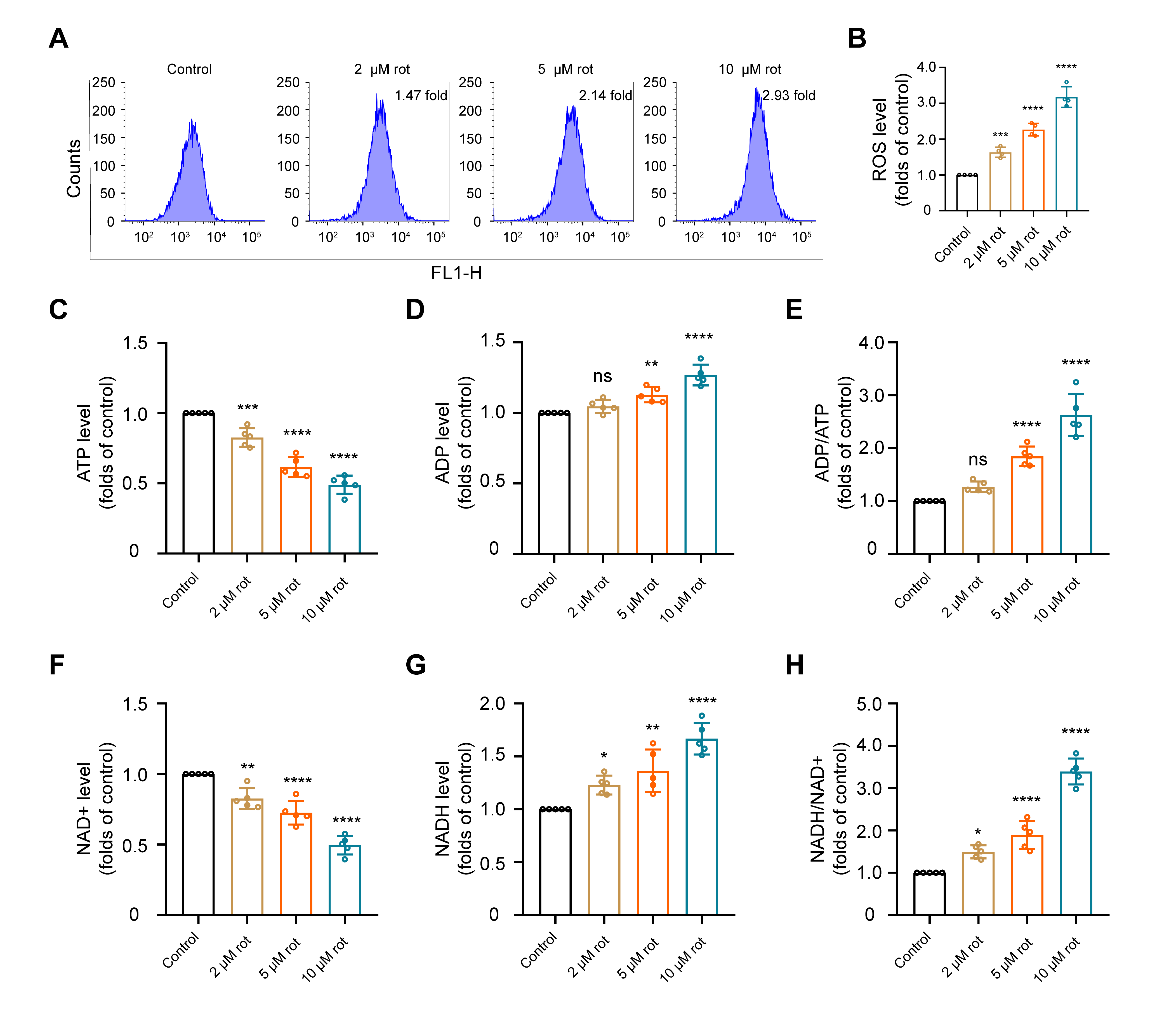


Supplemental Figure 3. Rot induces mitochondrial dysfunction in NPCs. (A) Flow cytometric analysis of ROS levels after exposure to different concentrations of rot. (B) Relative levels of ROS in NPCs. Means ± SD, n=4. (C-E) Histogram showing the levels of ATP, ADP, and ADP/ATP in NPCs exposed to rot. (F-H) Histogram showing the levels of NAD+, NADH, and NADH/NAD+ in NPCs exposed to rot. Means ± SD, n=5. *P < 0.05, **P < 0.01, ***P < 0.001, ****P < 0.0001, ns = not significant vs. the control group. BMSCs, bone marrow mesenchymal stem cells; NPCs, nucleus pulposus cells; rot, rotenone.


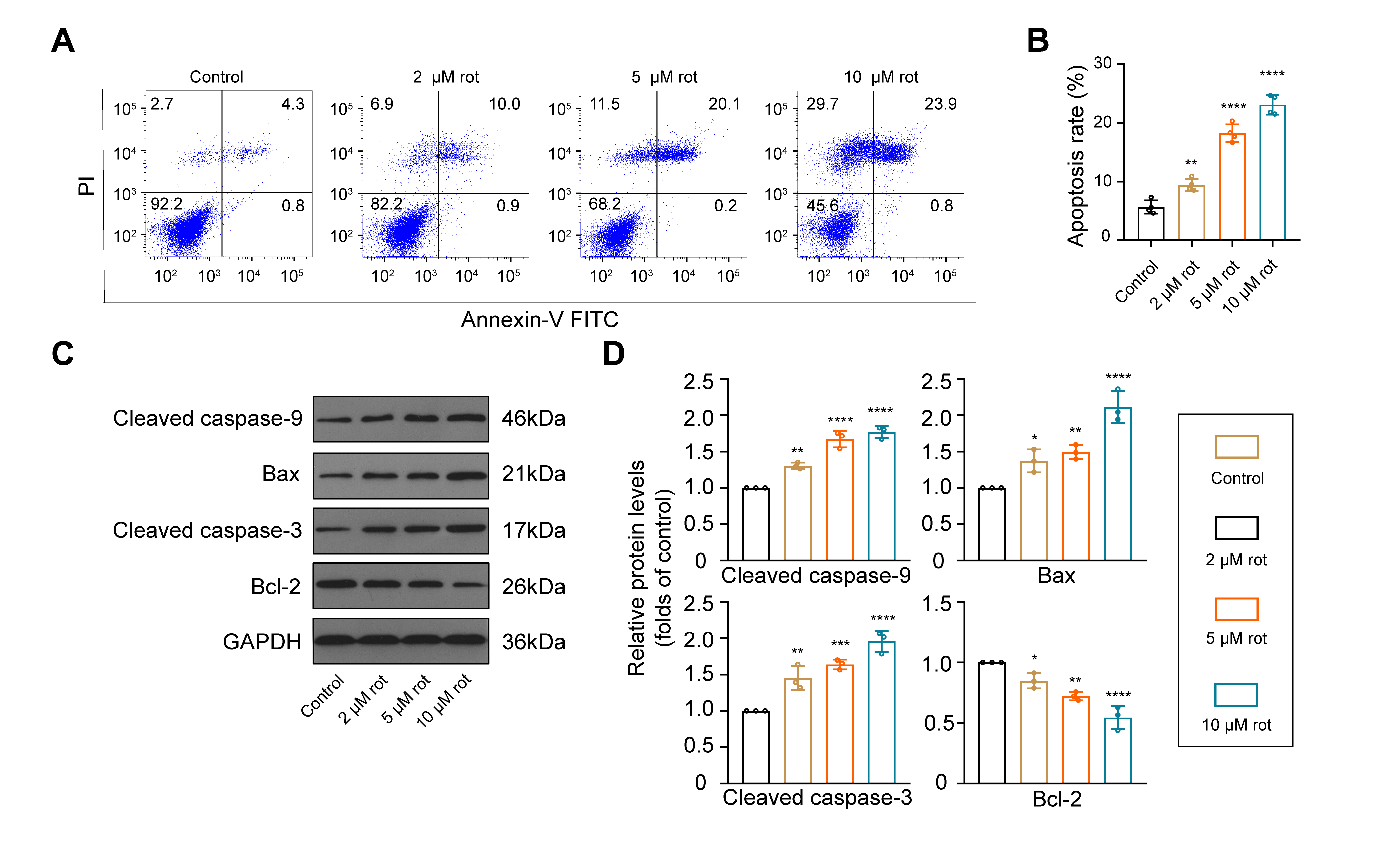


Supplemental Figure 4. Rot induces apoptosis in NPCs. (A) Flow cytometric analysis of the apoptosis rate after exposure to different concentrations of rot. (B) Histogram showing the apoptosis rate in NPCs. Means ± SD, n=4. (C) Representative Western blots showing the expression of cleaved caspase-9, Bax, cleaved caspase-3, and Bcl-2 in NPCs exposed to the indicated concentrations of rot. (D) Relative protein levels of cleaved caspase-9, Bax, cleaved caspase-3 and Bcl-2. Means ± SD, n=3. *P < 0.05, **P < 0.01, ***P < 0.001, ****P < 0.0001, ns = not significant vs. control group. BMSCs, bone marrow mesenchymal stem cells; NPCs, nucleus pulposus cells; rot, rotenone.


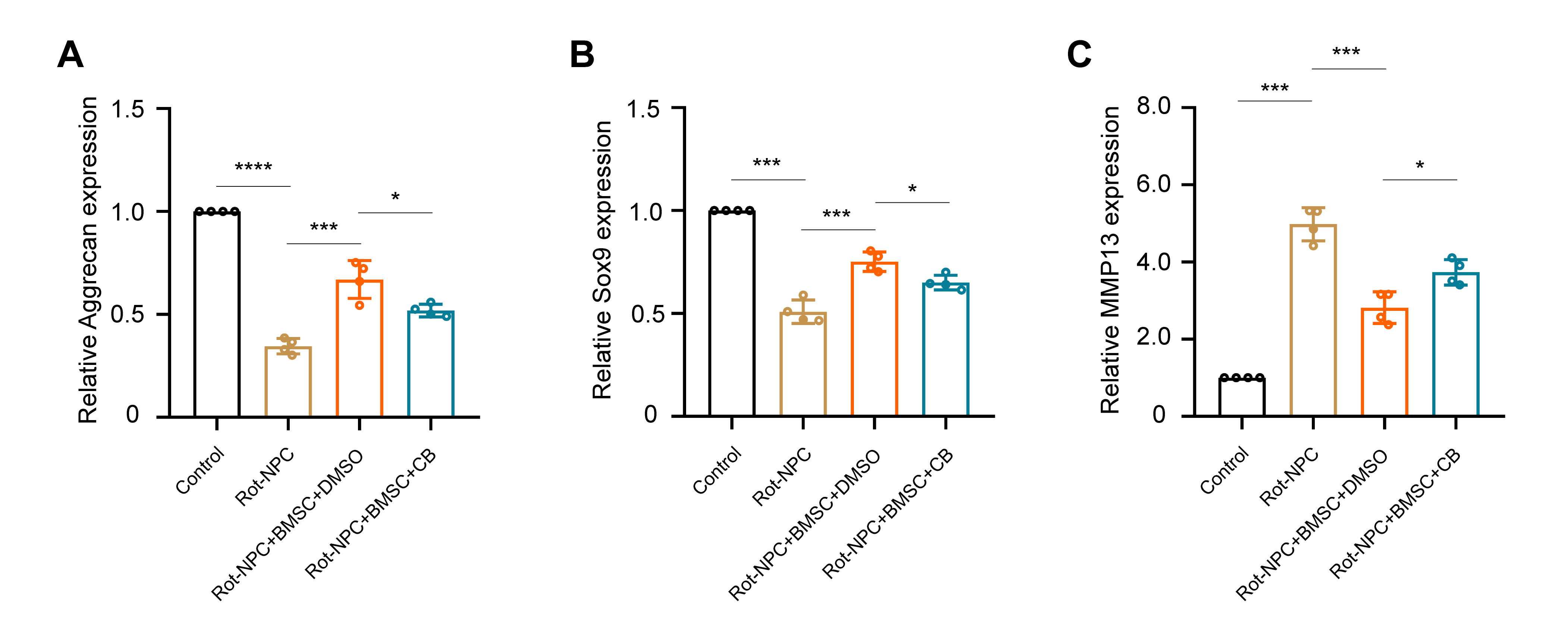


Supplemental Figure 5. The effects of mitochondrial transfer on matrix anabolism and catabolism in NPCs. (A-C) The mRNA expression levels of Aggrecan, Sox9, and MMP13. Means ± SD, n=4. *P < 0.05, ***P < 0.001, ****P < 0.0001. BMSCs, bone marrow mesenchymal stem cells; MMP13, matrix metalloproteinase 13; NPCs, nucleus pulposus cells; rot, rotenone.
